# Nonsuicidal self-injury, psychiatric disorders and pathological internet use among adolescents

**DOI:** 10.1192/j.eurpsy.2022.650

**Published:** 2022-09-01

**Authors:** G. Mészáros, D. Győri, L.O. Horváth, D. Szentivanyi, J. Balazs

**Affiliations:** 1 Semmelweis University, Faculty Of Medicine, Department Of Psychiatry And Psychotherapy, Budapest, Hungary; 2 Semmelweis University, Mental Health Sciences School Of Ph.d, Budapest, Hungary; 3 Eötvös Loránd University, Doctoral School Of Psychology, Budapest, Hungary; 4 Eötvös Loránd University, Institute Of Psychology, Budapest, Hungary; 5 Pedagogical Assistance Services, -, Budapest, Hungary; 6 Eötvös Loránd University, Institute Of Psychology, Department Of Developmental And Clinical Child Psychology, Budapest, Hungary; 7 Bjørknes University College, -, Oslo, Norway

**Keywords:** NSSI, PIU, psychiatric disorder, adolescent

## Abstract

**Introduction:**

Previous studies underline the importance of internalising disorders as risk factors of nonsuicidal self-injury (NSSI), meanwhile only a few research draw the attention to the role of externalising disorders. The possible association between NSSI and pathological internet use (PIU) is also understudied.

**Objectives:**

The purpose of this study was: 1) to investigate the frequency of NSSI among adolescents with different psychopathology and in different internet user groups of adolescents, 2) to understand the mediator role of psychiatric disorders between NSSI and PIU.

**Methods:**

Adolescents were enrolled from a clinical (Vadaskert Child Psychiatric Hospital, Budapest, Hungary) and a school based population (high schools in Budapest, Hungary). The used measurements were: Strengths and Difficulties Questionnaire (SDQ), Deliberate Self-Harm Inventory, Young Diagnostic Questionnaire for Internet Addiction, Mini International Neuropsychiatric Interview Kid.

**Results:**

There was significant difference in the frequencies of NSSI in SDQ subgroups (U=2127.000; z=-6.170; p <0.001). There was also significant difference in NSSI frequency between normal- and pathological internet users (U=2020.000; z=-2,501; p <0.017 p=0.012). According to the mediator model there was no direct association between PIU and NSSI, however it was mediated by different psychiatric disorders (affective disorders, anxiety disorders, attention deficit/hyperactivity disorder, conduct disorder, opposition defiant disorder, psychoactive substance abuse/dependence, psychotic disorders, suicidal behavior).

**Conclusions:**

The results strengthen that both internalising- and externalising psychopathology are associated with NSSI. Moreover this study underlines the importance of careful screening and treating of comorbid disorders with PIU, which can have a role in the prevention of NSSI and suicide as well.

**Disclosure:**

No significant relationships.

